# Supplementary figures and images for: A 3D-printed condom intrauterine balloon tamponade: Design, prototyping, and technical validation
Source: PLoS One. 2024 Jun 11;19(6):e0303844. doi: 10.1371/journal.pone.0303844 (PMC11166290; doi:10.1371/journal.pone.0303844)

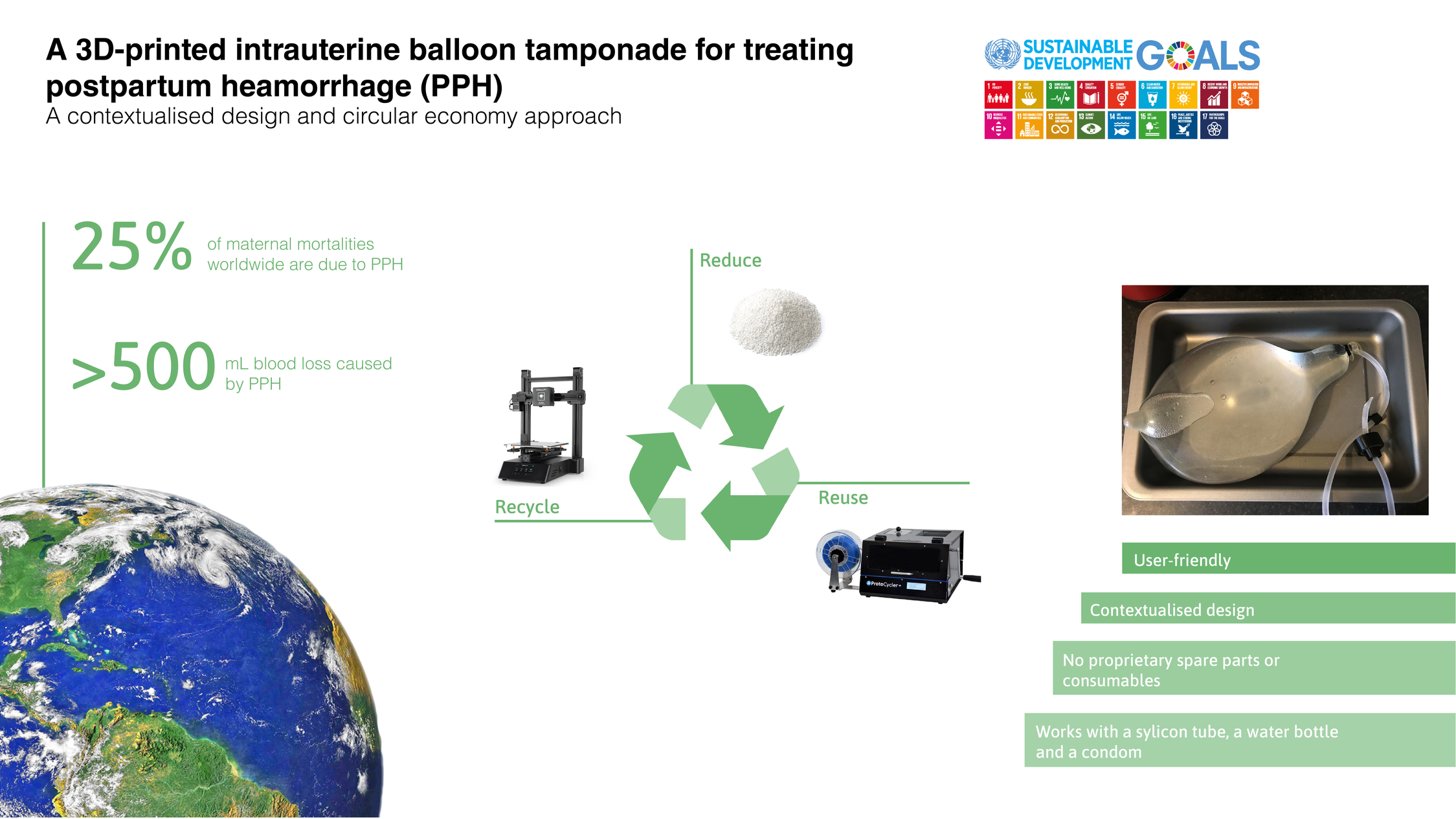

Supplement: S1 Fig — (TIF) [file pone.0303844.s001.tif]
