# Supplementary material for: A 3D-printed condom intrauterine balloon tamponade: Design, prototyping, and technical validation
Source: PLoS One. 2024 Jun 11;19(6):e0303844. doi: 10.1371/journal.pone.0303844 (PMC11166290; doi:10.1371/journal.pone.0303844)
Supplement: Attachment — Submitted filename: Piaggio_David 3D uterine balloon.docx [file pone.0303844.s003.docx]

This is very important development that should tested with real PPH cases, where the other management options like uterotonics are also exacting pressure on the uterus. Few comments for the authors to clarify

1. The abstract results are written as conclusion of the prototyping but not the results. Please state the results
2. Lines 2 and 3: Define the abbreviation LRSs and LMIC since they appear for the first time. There are several abbreviations that must be defined other than assuming that the readers know what it is.
3. You will a reference for this statement “Postpartum haemorrhage is defined as heavy bleeding after birth and it can be classed as “primary” if there is a 500-ml blood loss (*>*2 litres in case of severe haemorrhage) within the first 24 hours after birth, and as “secondary” if there is abnormal or heavy vaginal bleeding between 24 hours and 12 weeks after birth.”
4. Lines 18-20, the authors mention that the uterine balloon is among the first line management option, they need to check the citation well. It is for refractory management of PPH.
5. In line 82-83, The parts that underwent 3D printing were: 1) A valve 2) A flow-stopper 3) A modified bottle cap 4) A uterus model. What about the condom uterine balloon. It is not clear how this is different from ordinary condom
6. With several parts making the system, what were the different points of leakage of water.
7. Although the circular economic model is mentioned in the introduction and the discussion, the authors have not demonstrated how it applied in the methods and results. Which parts are re-usable.
8. What is the likely cost of the device
