# Supplementary material for: A 3D-printed condom intrauterine balloon tamponade: Design, prototyping, and technical validation
Source: PLoS One. 2024 Jun 11;19(6):e0303844. doi: 10.1371/journal.pone.0303844 (PMC11166290; doi:10.1371/journal.pone.0303844)
Supplement: Attachment — Submitted filename: Intrauterine balloon tamponade eview response.docx [file pone.0303844.s004.docx]

|  | **Comment** | **Reply** |
| --- | --- | --- |
| Editor | 1. Please ensure that your manuscript meets PLOS ONE's style requirements, including those for file naming. The PLOS ONE style templates can be found at <https://journals.plos.org/plosone/s/file?id=wjVg/PLOSOne_formatting_sample_main_body.pdf> and  <https://journals.plos.org/plosone/s/file?id=ba62/PLOSOne_formatting_sample_title_authors_affiliations.pdf> | Thank you for your valuable feedback.  The latex template was downloaded from the official website of PLOS one. If this is not ok, could you please share with us the template we could use? |
|  | ""Upon re-submitting your revised manuscript, please upload your study’s minimal underlying data set as either Supporting Information files or to a stable, public repository and include the relevant URLs, DOIs, or accession numbers within your revised cover letter. For a list of acceptable repositories, please see <http://journals.plos.org/plosone/s/data-availability#loc-recommended-repositories>. Any potentially identifying patient information must be fully anonymized.  Important: If there are ethical or legal restrictions to sharing your data publicly, please explain these restrictions in detail. Please see our guidelines for more information on what we consider unacceptable restrictions to publicly sharing data: <http://journals.plos.org/plosone/s/data-availability#loc-unacceptable-data-access-restrictions>. Note that it is not acceptable for the authors to be the sole named individuals responsible for ensuring data access.  We will update your Data Availability statement to reflect the information you provide in your cover letter | The study’s minimal underlying data is now submitted as supporting information. No ethical nor legal restrictions are present. |
|  | Please review your reference list to ensure that it is complete and correct. If you have cited papers that have been retracted, please include the rationale for doing so in the manuscript text, or remove these references and replace them with relevant current references. Any changes to the reference list should be mentioned in the rebuttal letter that accompanies your revised manuscript. If you need to cite a retracted article, indicate the article’s retracted status in the References list and also include a citation and full reference for the retraction notice. | The reference list has been reviewed and is complete and correct. |
| Reviewer 1 | The paper is addressing an important intervention component to manage PPH esp in low resource setting. However the technical aspects are not clear and comparison with available standard methods is not explicitly described. The contents of the UBT need to be described in detail what material, size, applicability and how this use is supposed to be different than others. | Thank you for your precious feedback.  Further information on the design was added, with specific regard to costs and reusability. Detailed drawings or sizes will be available by contacting the authors, upon reasonable request.  The novelty of our system has been stressed. |
|  | I do not agree with the advantage of being able to sterilise this UBT as there are multiple low cost dispensable UBTs available. Hence its not clear to the reader how this UBT is better in either the contents, material used, ease of use, capacity of the device to hold the fluid, duration that could be kept in the uterus. | The re-use of parts of this UBT with the possibility of recycling previously used plastics and components of this device is one of the core advantages.  According to our field studies and literature search, consumables are one of the bottlenecks when it comes to low resource settings. UBTs have similar challenges. We agree that this might not be the case for all low resource settings, but it is a widespread challenge that justifies our project. This novel approach inspired by frugal and circular practices would also benefit the sustainability of the healthcare sector. This is now a very hot topic, as heralded by the sustainable development goals of the United Nations. |
|  | The figure of inflated balloon also does not visually satisfy that it takes shape of the uterus such that its entire inner surface gets pressure from the inflated balloon | The figure is now modified accordingly. |
|  | I suggest the paper be reviewed by biomedical engineer expert to understand the nitty-gritties. Every device has to meet an international quality standard (ISO) . There is no mention how this device falls within those standards | We are happy for the paper to be reviewed by a fellow biomedical engineer.  Our regulatory affairs work is now clarified better in the text (intro) with the addition of a statement in the limitations under the discussions section.  Please notice that ours is a concept prototype and final adherence with standards is foreseen for higher technology readiness levels. |
|  | Authors must also be aware that 3 D printers may not be easily available in Low resource settings for which this device is being developed. | This is a fair concern. We believe that the collaboration with ad hoc centres perhaps based in large hospitals or at university could really support this change of paradigm, towards the support of local producers and self-sustainability. |
| Reviewer 2 | This is very important development that should tested with real PPH cases, where the other management options like uterotonics are also exacting pressure on the uterus. Few comments for the authors to clarify | Thank you for your precious feedback.  The next step for this kind of prototype would be in vivo comparison with other treatments. However, due to ethics limitations and the different nature of the current study (pre-clinical validation), this is not included in the remit of this publication. |
|  | The abstract results are written as conclusion of the prototyping but not the results. Please state the results. | This has now been corrected. |
|  | Lines 2 and 3: Define the abbreviation LRSs and LMIC since they appear for the first time. There are several abbreviations that must be defined other than assuming that the readers know what it is. | The abbreviations are now defined when first used. |
|  | You will a reference for this statement “Postpartum haemorrhage is defined as heavy bleeding after birth and it can be classed as “primary” if there is a 500-ml blood loss (>2 litres in case of severe haemorrhage) within the first 24 hours after birth, and as “secondary” if there is abnormal or heavy vaginal bleeding between 24 hours and 12 weeks after birth.” | A reference has been added.  https://www.ncbi.nlm.nih.gov/books/NBK499988/ |
|  | Lines 18-20, the authors mention that the uterine balloon is among the first line management option, they need to check the citation well. It is for refractory management of PPH. | This has been modified. |
|  | In line 82-83, The parts that underwent 3D printing were: 1) A valve 2) A flow-stopper 3) A modified bottle cap 4) A uterus model. What about the condom uterine balloon. It is not clear how this is different from ordinary condom | The condom indeed is an ordinary condom. This is the point of strength of our design as specified in the section “other parts and assembly of the systems”:  *Among the other parts, a common polyethylene terephthalate (PET) 500 mL bottle was used as a water reservoir, a standard latex condom was used as an inflatable balloon tamponade and a standard silicon tube (with an external diameter of 6 mm, 50 cm in*  *length) was used for channeling water in the balloon.*  For this reason, we deemed there was no need for specifying it in other sections. |
|  | With several parts making the system, what were the different points of leakage of water. | Points of leakage are now specified. |
|  | Although the circular economic model is mentioned in the introduction and the discussion, the authors have not demonstrated how it applied in the methods and results. Which parts are re-usable. | This is now better specified in the methods and results. |
|  | What is the likely cost of the device | A cost estimate is given in the text with motivation. |
