# Supplementary material for: A 3D-printed condom intrauterine balloon tamponade: Design, prototyping, and technical validation
Source: PLoS One. 2024 Jun 11;19(6):e0303844. doi: 10.1371/journal.pone.0303844 (PMC11166290; doi:10.1371/journal.pone.0303844)
Supplement: Attachment — Submitted filename: Reply to comments.docx [file pone.0303844.s005.docx]

| **Reviewer** | **#** | **Comment** | **Reply** |
| --- | --- | --- | --- |
| **Editor** | **1** | Your manuscript has been evaluated by two reviewers: one from the previous round (Reviewer 1) and one new reviewer with expertise in biomedical engineering (Reviewer 3). Their comments are appended below.  Reviewer 1 is satisfied with the changes you have made. Reviewer 3 recognizes the changes, but has stated that clinical experiments and a comparison with standard methods are necessary to support the conclusions of this study.    Recognizing that this work was intended as a pre-clinical investigation, clinical validation will be a necessary step, but reasonable to consider outside the scope of the current study. However, demonstration of performance against existing comparable balloon tamponades would help validate the claim that this newly proposed concept has comparable (or at least practical given the other stated advantages) functionality. Please also ensure that any claims of potential efficacy are appropriately qualified with statements clarifying that no clinical experiments have been carried out. | **Thank you for your feedback. We appreciate your suggestion and we have done our best to demonstrate the device's performance against existing comparable ones.**  **We have also added a new section (both in the methods and results) for comparison with other devices, based on a proactive FMEA risk assessment.**  **Please find our changes in blue font in the text.** |
| **Rev1&3** | **2** | - **All comments from previous rounds have been addressed.** - The manuscript is technically sound, and the data support the conclusions: yes and partly. - The statistical analysis has been performed appropriately and rigorously: N/A and No. - **The authors made all data** **underlying the findings in their manuscript fully available.** - The manuscript is presented in an intelligible fashion and written in standard English. | **Thank you for your valuable feedback. Please find below our reply to your comments and the changes in the text written in blue color.** |
| **Rev3** | **3** | I really appreciate all your hard work to write this article. however, it still needs more efforts to finalize it. A set of clinical experiments is needed to validate the proposed catheter. Also, a comparison with one of the standard methods is highly required to support the concluded results.  best regards | **Thank you for your feedback. Please see our changes in blue font in the text. We have also added a new section (both in the methods and results) for further comparison with other devices, based on FMEA.**  **We agree that a set of clinical experiments would be very useful, unfortunately the device is not at that stage yet. A clinical validation is therefore deemed out of scope for this work, but we have added a new section (both in the methods and results) for comparison with other devices, based on a proactive FMEA risk assessment, to compare our approach to standard methods.** |
