# Supplementary material for: A 3D-printed condom intrauterine balloon tamponade: Design, prototyping, and technical validation
Source: PLoS One. 2024 Jun 11;19(6):e0303844. doi: 10.1371/journal.pone.0303844 (PMC11166290; doi:10.1371/journal.pone.0303844)
Supplement: Attachment — Submitted filename: Third round of review replies IUB PLOS One.docx [file pone.0303844.s006.docx]

Reply to comments

| ID | Comment | Reply |
| --- | --- | --- |
| 1- Editor | Dear authors, please correct the manuscript by separating the single paragraph of the discussion into 3 or 4 and leave it in accordance with the following reviewers' recommendations: "this version of the manuscript has more typos than the previous one (e.g. periods in titles of some subsections of the " Results" section and the "Discussions and Conclusions" section consists of only one long paragraph). Also, the reference section needs to be revised carefully (e.g. ref 23 and 24 are repeated in 37 and 38).", and "the quality of Figure 1 must be improved and the Figure 2 showing the complete prototype, including the used pump." | Dear Editor, Many thanks for your valuable comments.  Both the introduction and discussion were divided into paragraphs. Typos were corrected. References were corrected. Figure 1 is already exported in 300dpi, quality cannot be improved. It is perhaps possible that the reviewer has seen the compressed image that is added in the journal compiled PDF. Figure 2 was retaken and uploaded.  Due to the nature of the changes being made (e.g., paragraphing), there is no markup version. |
| Reviewers |  | Thank you for your valuable comments.  The editor summarised the required changes (see above). We managed to modify the article accordingly and to reply as above. Thanks. |
